# Antineutrophil cytoplasmic antibody (ANCA)-associated autoimmune diseases induced by antithyroid drugs: comparison with idiopathic ANCA vasculitides

**DOI:** 10.1186/ar1789

**Published:** 2005-07-13

**Authors:** Branka Bonaci-Nikolic, Milos M Nikolic, Sladjana Andrejevic, Svetlana Zoric, Mirjana Bukilica

**Affiliations:** 1 Institute of Allergy and Clinical Immunology, Clinical Centre of Serbia, Belgrade, Serbia and Montenegro; 2 Institute of Dermatology, Clinical Centre of Serbia, Belgrade, Serbia and Montenegro; 3 Institute of Endocrinology, Clinical Centre of Serbia, Belgrade, Serbia and Montenegro; 4 Institute of Rheumatology, Belgrade, Serbia and Montenegro

## Abstract

Clinical and serological profiles of idiopathic and drug-induced autoimmune diseases can be very similar. We compared data from idiopathic and antithyroid drug (ATD)-induced antineutrophil cytoplasmic antibody (ANCA)-positive patients. From 1993 to 2003, 2474 patients were tested for ANCA in the Laboratory for Allergy and Clinical Immunology in Belgrade. Out of 2474 patients, 72 (2.9%) were anti-proteinase 3 (PR3)- or anti-myeloperoxidase (MPO)-positive and their clinical and serological data were analyzed. The first group consisted of ANCA-associated idiopathic systemic vasculitis (ISV) diagnosed in 56/72 patients: 29 Wegener's granulomatosis (WG), 23 microscopic polyangiitis (MPA) and four Churg-Strauss syndrome. The second group consisted of 16/72 patients who became ANCA-positive during ATD therapy (12 receiving propylthiouracil and four receiving methimazole). We determined ANCA and antinuclear (ANA) antibodies by indirect immunofluorescence; PR3-ANCA, MPO-ANCA, anticardiolipin (aCL) and antihistone antibodies (AHA) by ELISA; and cryoglobulins by precipitation. Complement components C3 and C4, alpha-1 antitrypsin (α1 AT) and C reactive protein (CR-P) were measured by nephelometry. Renal lesions were present in 3/16 (18.8%) ATD-treated patients and in 42/56 (75%) ISV patients (*p *<0.001). Skin lesions occurred in 10/16 (62.5%) ATD-treated patients and 14/56 (25%) ISV patients (*p *<0.01). ATD-treated patients more frequently had MPO-ANCA, ANA, AHA, aCL, cryoglobulins and low C4 (*p *<0.01). ISV patients more frequently had low α1 AT (*p *= 0.059) and high CR-P (*p *<0.001). Of 16 ATD-treated patients, four had drug-induced ANCA vasculitis (three MPA and one WG), while 12 had lupus-like disease (LLD). Of 56 ISV patients, 13 died and eight developed terminal renal failure (TRF). There was no lethality in the ATD-treated group, but 1/16 with methimazole-induced MPA developed pulmonary-renal syndrome with progression to TRF. ANCA-positive ISV had a more severe course in comparison with ATD-induced ANCA-positive diseases. Clinically and serologically ANCA-positive ATD-treated patients can be divided into two groups: the first consisting of patients with drug-induced WG or MPA which resemble ISV and the second consisting of patients with LLD. Different serological profiles could help in the differential diagnosis and adequate therapeutic approach to ANCA-positive ATD-treated patients with symptoms of systemic disease.

## Introduction

Antineutrophil cytoplasmic antibodies (ANCA) specific for proteinase 3 (PR3) and myeloperoxidase (MPO) are associated with necrotizing vasculitides, especially Wegener's granulomatosis (WG), microscopic polyangiitis (MPA) and idiopathic crescentic glomerulonephritis [1]. Pathogenesis of ANCA-associated idiopathic systemic vasculitides (ISV) is not well understood, but it has been shown that ANCA-activated neutrophils contribute to oxidative and proteolytic damage of blood vessels [2]. Cytoplasmic PR3-ANCA has high specificity (99%) for the newly diagnosed WG [3]. Perinuclear MPO-ANCA is a good serological marker for MPA, but it can also be found in patients with systemic lupus erythematosus (SLE), rheumatoid arthritis, drug-induced vasculitides (DIV), etc [4].

ANCA-associated ISV are rare and their annual incidence is approximately 9.5 per million (in Germany) [3]. Although WG and MPA belong to the ISV group, they can be triggered by some chemicals, viral and bacterial infections and certain drugs, among which antithyroid drugs (ATDs) are very common [5]. Propylthiouracil (PTU) and methimazole (MM) may induce ANCA-positive vasculitides [6].

The clinical and serological profiles of idiopathic and drug-induced autoimmune diseases (DIDs) can be very similar. Contrary to idiopathic vasculitides, DIDs have a milder course and often do not necessitate cytotoxic drug therapy [5]. Pathogenesis and clinical/serological characteristics of ANCA-associated diseases triggered by ATD have not been sufficiently investigated. In a retrospective study, we compared data from idiopathic and ATD-induced ANCA-positive patients.

## Patients and methods

### Patients

From 1993 to 2003, 2474 patients were tested for ANCA in the Laboratory for Allergy and Clinical Immunology in Belgrade, and 72/2474 (2.9%) were PR3-ANCA or MPO-ANCA positive. The maximal follow-up period was 11 years and the minimal was 6 months, while the median follow-up time was 4.5 years.

PR3-ANCA- and MPO-ANCA-positive patients were divided into two groups. The first group consisted of ANCA-associated ISV that was diagnosed in 56/72 (77.7%) patients (29 WG, 23 MPA and four Churg-Strauss syndrome) according to Chapel Hill Consensus Conference [7]. Disease activity was assessed according to the Birmingham Vasculitis Activity Score (BVAS) [8]. A biopsy was taken from 47/56 patients (biopsies were not performed in the other nine patients due to poor/critical general condition). Kidney biopsy was performed in 38 patients (25 segmental necrotizing glomerulonephritis (SNGN) with cellular and fibrous crescents, four SNGN without crescents, six SNGN with arteritis and three mesangial proliferation). Lung biopsy was performed in 10 patients (four granulomatous inflammation with multinucleated giant cells with foci of neutrophils, leucocytoclasia and necrosis; two hemorrhagic alveolar capillaritis with septal infiltration of neutrophils and necrosis; and four perivascular hemorrhage with mixed infiltrate composed of neutrophils and mononuclear cells). Skin biopsy was performed in six patients (four leucocytoclastic vasculitis and two swollen endothelial cells, neutrophil infiltration without frank fibrinoid necrosis); nasal lesions in four (one giant cell granuloma and three mucosal neutrophil infiltration); and lymph nodes in one patient (perivascular infiltration of neutrophils and lympho/monocytes). Complete blood count, renal function tests, proteinuria and urine examination were done at the time of diagnosis and then serially during the follow-up period. Initially all patients were treated with cyclophosphamide (six intravenous (iv) pulses at 700 mg/m^2 ^or 2 mg/kg/day orally) in combination with prednisone at 1 mg/kg/day in most patients. A few patients with milder disease received cyclophosphamide with prednisone at 0.5 mg/kg/day.

The second group, 16/72 (22.3%), consisted of ANCA-positive patients who developed symptoms of systemic disease during ATD therapy. The diagnosis of Graves' disease (GD) and Hashimoto thyroiditis (HT) was made according to established criteria [9]. One patient with GD was in the fifth month of pregnancy, one had Pluriglandular Autoimmune Syndrome type II and the only male person with GD was in remission from IgA nephropathy. Of the 16 patients, six were taking PTU for a relapse of hyperthyroidism. The majority of patients (9/16) had a maintenance therapy with PTU at 50–150 mg/day or with MM at 5–15 mg/day. Florid hyperthyroidism was present in 7/16 individuals, and these patients were treated with 200–400 mg/day of PTU or with 15–40 mg/day of MM. Biopsy was taken from 14/16 ATD-treated ANCA-positive patients. Skin biopsy was performed in 10 patients (eight leucocytoclastic vasculitis with fibrinoid necrosis and two swollen endothelial cells with edema), kidney biopsy in two (two SNGN with fibrous crescents and one SNGN without crescents) and lung biopsy in two (one granulomatous inflammation with focus of neutrophils, leucocytoclasia and necrosis and one perivascular mixed infiltrate composed of neutrophils and mononuclear cells). Immunosuppressive therapy was given to 6/16 ATD-treated patients (corticosteroids in three and cyclophosphamide iv pulses or orally in three). A pregnant patient with extensive cutaneous necroses was treated with intravenous methylprednisolone pulses (three pulses of 500 mg) followed by oral prednisone at 0.5 mg/kg with gradual dose tapering.

### Methods

All samples were obtained at the time of diagnosis and none of the patients had previously received immunosuppressive treatment. IgG ANCA titers were determined at the initial visit and at least 6 months thereafter using an indirect immunofluorescence (IIF) assay with 'in-house' ethanol-fixed neutrophil preparation as previously described, starting with a titer of 1:16 [10]. PR3-ANCA and MPO-ANCA specificity (cut-off 15 U/ml) was confirmed by direct ELISA (Varelisa; Pharmacia Upjohn Diagnostics, Freiburg, Germany).

Antinuclear (ANA) IgG antibodies were detected by IIF using HEp 2 cells (Mast Diagnostica, Reinfeld, Germany). Anti-dsDNA IgG antibodies were detected by IIF on *Crithidia lucilliae *(Mast Diagnostica). The concentration of specific antibodies (RNP, Sm, SS-A, SS-B, Scl 70, CENP and Jo) in ANA-positive sera were measured (cut-off 8 U/ml) by commercial direct ELISA (Varelisa).

Concentrations of anticardiolipin antibodies (aCL) IgG and IgM, anti-beta (β)-2 glycoprotein (GP) I, IgG and IgM and anti-histone antibodies (AHA) were measured by commercial direct ELISA (Varelisa). Antimicrosomal and antithyreoglobulin IgG antibodies were detected by IIF using primate thyroid gland as substrate, starting with a titer of 1:20. Concentrations of C3, C4 component complement, C reactive protein (CR-P) and alpha-1 antitrypsin (α1 AT) were measured by nephelometry (Orion Diagnostica, Espoo, Finland). Cryoglobulins were investigated by the standard procedure [11]. Cryoprecipitate was analyzed for detection of ANCA by IIF, rheumatoid factor by nephelometry (Orion Diagnostica) and the presence of paraprotein by immunofixation (Binding Site, Birmingham, UK). Positive sera were analyzed for the presence of hepatitis C virus by ELISA test (Biokit, Barcelona, Spain).

### Statistical analysis

Frequencies of non-parametric characteristics in ISV and DID groups were compared using the χ^2 ^test or Fisher's exact test. Student's *t*-test was applied in comparisons of continuous variables. Correlations were expressed using Spearman's rank correlation coefficient. Probability (*p*) values less than 0.05 were considered statistically significant. Data were analyzed with SPSS statistical software v10.00 for Windows (SPSS, Inc, Chicago, IL, USA).

### Ethics

Informed consent was obtained from each patient and the Ethics Committee of the Clinical Center of Serbia approved the study protocol.

## Results

In the ATD-associated MPO- or PR3-ANCA-positive group, there were statistically more women than men (*p *<0.01) and patients were significantly younger (*p *<0.001) in comparison with patients with ISV (Table 1). The average symptom duration before diagnosis was significantly shorter in ATD-treated patients (*p *<0.001). Mean treatment duration with ATD was 20 ± 6 months (range 6–36 months): PTU was given 25 ± 7 and MM 11 ± 4 months before symptoms occurred. ATDs were given for longer than the usual 18 months in 12/16 patients. Most of patients in the DID group had GD (81%) and were treated with PTU (75%).

Idiopathic and ATD-induced ANCA-positive patients were characterized by a high frequency of arthralgia and myalgia. On the other hand, ISV patients more frequently had fever, weight loss, renal manifestations, acute renal failure (ARF), pulmonal manifestations, pulmonary-renal syndrome, ear/nose and nervous system manifestations (Table 2).

In the ATD-treated group, only 1/16 patients developed ARF (SNGN with crescents) with severe pulmonary-renal syndrome; 2/16 patients developed hematuria and non-nephrotic range proteinuria. Only one female patient treated with MM had nodular lung infiltration pathohistologically verified as granulomatous vasculitis.

In contrast to patients with ISV, ATD-associated ANCA-positive patients had skin manifestations more frequently, especially urticaria-like vasculitis and urticaria (Table 2). Patients with urticaria-like vasculitis and purpura histologically had leucocytoclastic vasculitis. A pregnant patient with poorly controlled GD had extensive cutaneous necroses and hemorrhagic bullae, while a patient with pluriglandular autoimmune syndrome had lower leg ulcerations. Digital gangrenes and mouth ulcerations were noted only in PR3-ANCA-positive ISV patients.

None of the ATD-treated patients manifested central nervous system effects, while 1/16 patients had predominantly peripheral sensory polyneuropathy that regressed upon drug withdrawal.

ATD-induced ANCA-positive patients more frequently had MPO-ANCA, ANA, AHA, IgM aCL, concomitant presence of both IgM and IgG aCL, IgM anti-β2 GP I, cryoglobulinemia and decreased C4 (Table 3). Demonstration of pANCA and cANCA by IIF correlated with MPO-ANCA and PR3-ANCA positivity in ELISA, in all except one patient with WG, in whom cANCA had MPO specificity. In all ANA-positive patients, ANCA/ANA titer proportion was >2. In the group of ATD-associated ANCA-positive patients, 1/16 had anti-DNA antibodies (titer 1:80), while 1/16 had anti-SSA (an antibody associated with Sjogren's Syndrome) in a concentration of 30.5 U/ml. Decreased C4 was found in three individuals with cryoglobulinemia, two of whom had ANA and leucopenia. None of the three ATD-treated patients with cryoglobilinemia had hepatitis C virus infection and no rheumatoid factor or paraproteins were detected in cryoprecipitate. Perinuclear ANCA was present in cryoprecipitate of one patient. Both patients with extensive cutaneous necroses had high MPO-ANCA concentrations, type III cryoglobilinemia, IgM aCL and slightly decreased C4.

Analysis of clinical and serological parameters revealed that the ATD-associated ANCA-positive patients can be divided into two groups (Table 4). The first group (4/16 patients) had clinical and serologic parameters similar to patients with ISV. Among these patients, one had WG and three had MPA (Table 5). The second group (12/16 patients) had clinical and serologic parameters typical for MPO-ANCA-associated lupus-like disease (LLD) [9]. No patient fulfilled criteria for SLE [12] and primary antiphospholipid syndrome - APS [13].

In the ISV group, at the beginning of the disease, there was no correlation between cANCA or pANCA titers and BVAS (*p *> 0.05). Also, in drug-induced MPA patients (Table 5), there was no correlation between pANCA titers and BVAS (*p *> 0.05).

All patients with ISV were treated with immunosuppressive therapy. In ATD-treated patients, only 6/16 patients were treated with immunosuppressive therapy (Table 5). Although clinical remission was achieved in all 16 ATD-treated patients after 6 months, ANCA titers slowly declined and only finally disappeared after 2 years. Also, clinical remission preceded the disappearance of organ-nonspecific antibodies. Anti-DNA, AHA and cryoglobulins disappeared in 6 months, while aCL and anti-β2 GP I concentrations declined, but persisted for up to 1.5 years. Clinical remission preceded the disappearance of ANCA in 6/16 patients who were treated with immunosuppressive drugs.

In patients with ISV, induction of clinical remission correlates with significant ANCA titers reduction (Table 5), and this was observed in 46/56 individuals who were not resistant to standard immunosuppressive therapy. The standard regimen could not induce clinical remission and ANCA titer reduction in 10/56 patients, necessitating introduction of additional immunosuppressive therapy including plasmapheresis in three and intravenous immunoglobulins (2 g/kg) in seven patients, respectively. No patient in the ATD-treated group had to be treated with additional immunosuppressive therapy.

In the ISV group, chronic renal failure was statistically more frequent (*p *< 0.01) than in ATD-treated ANCA-positive patients (Table 5). There were no differences in terminal renal failure (*p *> 0.05) between the groups. In 2/16 ATD-treated patients, the drugs (MM 20 mg/day and PTU 250 mg/day, respectively) were not discontinued in time, and they developed terminal renal failure and chronic renal failure, respectively. Both patients had ATD-induced MPA during a relapse of GD. ATD-associated LLD is characterized by a milder course and better prognosis than ATD-associated MPA (Table 5).

In the ISV group, lethal outcome was more frequent (*p *= 0.059) than in the DID group. In the ISV group, 6/56 patients died during the first episode, while out of the remaining 50, 47 had at least one relapse. In the ATD-treated group there were no disease relapses.

All the patients with ATD-induced disease are now in remission without any therapy, whilst the 37/43 patients with ISV need to receive maintenance therapy (*p *< 0.001).

After the ATD withdrawal, hyperthyroid patients were treated with radioactive iodine or subtotal thyroidectomy. In a pregnant woman with GD, potassium perchlorate was prescribed at the lowest dose, keeping the patient mildly hyperthyroid. A healthy child was born normally 3 months later.

## Discussion

Standard clinical and serologic criteria for discrimination of ANCA-associated ISV from DID are inadequate at the initial presentation. The histopathology can be inconclusive, since both entities are characterized by leucocytoclasia and fibrinoid necrosis of the blood vessels [14]. ANCA-positive DID can be 'pauci-immune' but also with strong vascular immune deposits [1].

In this 11-year retrospective study, we compared clinical and serological data from idiopathic and ATD-induced ANCA-positive patients. A higher number of younger females in the ATD-treated group was found (Table 1), because hyperthyroidism is predominantly a disease of this sex and age group [9]. On the other hand, WG and MPA are predominantly diagnosed in the older population [1]. ANCA-positive ATD-induced diseases occur more frequently in GD than in HT [6], which we also found (Table 1).

All ATDs can induce asymptomatic production of ANCA, but PTU most often induces ANCA-associated MPA or LLD [15]. In our study, apart from long-term ATD use [16], the repeated use of ATDs was also a risk factor for developing the disease. As PTU is more commonly associated with ANCA-positive DID, MM may be the preferred medication if hyperthyroidism is combined with other autoimmune diseases [17]. At the molecular level, PTU and MM are based on thionamide, and the substitution of one with the other should be undertaken with caution. On the other hand, patients should be promptly brought to euthyroid state. Since MM is contraindicated in pregnancy, PTU-induced disease in pregnancy demands cautious use of iodides [18]. This enabled a successful delivery in one of our patients.

The analysis of the clinical parameters (Table 2) showed that the first symptoms (arthralgias and myalgias) of ANCA-positive ISV and ATD-treated patients were similar. Shorter duration of the symptoms before diagnosis (Table 1) in ATD-treated patients can be explained by a higher percentage of early skin manifestations. Although arthralgias, myalgias and weight loss can be seen in hyperthyroidism, temperature peaks over 38.5°C, and renal and pulmonal involvement are unusual [9]. Sera *et al*. [19] followed 21 MPO-ANCA-positive patients treated with PTU: 12 had no symptoms but nine patients complained of myalgia, arthralgia or common cold-like symptoms after the appearance of MPO-ANCA.

ANCA-associated pulmonary-renal syndrome is the most severe presentation of necrotizing vasculitis. In ATD-treated patients, renal lesions may be mild but also fulminant, including development of ARF [20]. One MM-treated patient developed drug-induced WG, which is a very rare form of DID [21]. After discontinuing ATD, our patient was treated orally with cyclophosphamide and prednisone for 3 months, while Pillinger *et al*. induced remission of WG only by discontinuing PTU [21].

Cutaneous changes in ANCA-positive ATD-treated patients can vary enormously from maculopapular exanthema to pyoderma gangrenosum, cutaneous ulcerations and vesiculo-bullous lesions typical for bullous SLE [22,23]. Of our patients, 7/16 had urticaria and urticaria-like vasculitis while two had extensive cutaneous necrosis (Table 2). Urticaria-like vasculitis is frequently seen in patients with hypo-complementemic vasculitis and SLE. Gangrene and cutaneous necroses are poor signs of PR3-ANCA-positive ISV [24]. However, cutaneous necroses in our two ATD-treated ANCA-positive patients were not associated with a worse prognosis.

An analysis of the serologic profile of the ATD-treated patient group showed simultaneus presence of various autoantibodies (Table 3). In patients with GD and HT, even before starting ATD, different ANA were found [25], as well as different organ-specific autoantibodies.

On the other hand, most patients with GD and HT who were not treated with ATD do not have ANCA [19]. In the PTU-treated group, 25% of the patients had MPO-ANCA, while in the MM group only 3.4% had MPO-ANCA [15]. These findings suggest that the altered immune environment associated with GD and HT is not sufficient to develop ANCA, but treatment with ATD induces ANCA production.

The titer of pANCA was much higher in the ATD-treated group than in the ISV group, while concentrations of MPO-ANCA did not differ (Table 3). We suppose that patients taking ATD have ANCA specific to elastase and lactoferrin, which was described in PTU-induced vasculitis and LLD [26]. PTU is also associated with simultaneous production of MPO-ANCA and PR3-ANCA [26], which however, we did not see in any of our patients. It was shown that WG patients during ATD therapy changed PR3-ANCA to MPO-ANCA with flares of vasculitis [27].

The pathogenic role of ANCA remains controversial in ISV and DID. Because not all ANCA-positive patients develop the disease, some authors are considering, along with ATD, an infection as an additional trigger in inducing autoimmune diseases [20]. In our series of ATD-associated ANCA-positive patients, there were no clinical or laboratory parameters for infection. However, an unnoticed mild viral infection might induce priming (TNF-alfa) of neutrophils and translocation of PR3 and MPO to the cell surface [2,28].

Some authors are considering the possibility of cross-over epitopes between thyroid peroxidase and MPO [29]. We showed that only some MPO-ANCA-positive patients have antimicrosomal antibodies (Table 3). Besides 44% homology in amino acid sequences, there is no cross-reactivity between thyroid peroxidase and MPO [30].

On the other hand, it was shown that PTU accumulates in neutrophils, binds to MPO and induces production of cytotoxic products [31,32]. It has been suggested that some drugs could induce neutrophil apoptosis [33]. Moreover, neutrophil apoptosis, in the absence of priming, is associated with translocation of ANCA antigens to the cell surface [34]. Simultaneous presence of various autoantibodies (anti-DNA, AHA, anti-SSA, aCL and anti-β2 GP I) suggest that apoptotic blebs of neutrophils could be a source of immunogens in our patients with ATD-induced LLD [35,36].

ATD-induced ANCA-positive diseases are divided in two groups (Table 4). The first group (4/16) consisted of PR3- or MPO-ANCA-positive drug-induced WG or MPA which resemble ISV. The second group (12/16) consisted of MPO-ANCA-positive patients with LLD. We speculate that different clearance of apoptotic materials by macrophages might determine type of autoimmune response [37]. PTU-induced LLD was described in a patient with HLA DR9 haplotype whose twin sister had SLE [38].

Our ATD-associated LLD patients, despite high concentration of MPO-ANCA, had mild clinical courses. IgM anti-MPO antibody-secreting lymphocytes are present in the peripheral repertoire of lupus-prone mice but rarely differentiate into IgG-producing cells [39]. We can suppose that ATDs induce production of the potentially pathogenic MPO-ANCA IgG_4 _subclass [40]. MPO has the characteristics of a SLE-related antigen [39]. MPO-ANCA in SLE is most often found in the context of secondary APS [41], for which there were no criteria in our patients. Anti-β2 GP I antibodies are typical markers for APS [13] and have not been reported in ATD-induced ANCA-positive diseases. These data may have an impact on the approach to the patients since thrombotic events can be expected in case of an infection or a surgical procedure.

Simultaneous presence of cryoglobulins, aCL and anti-β2 GP I antibodies [42] in our two MPO-ANCA-positive patients with LLD synergistically contributed to development of cutaneous necrosis. However, we can assume that in patients with ISV, absolute or relative deficiency of α1 AT (Table 3), which is a specific PR3 inhibitor, could play a role in necrosis. The clinical picture of ISV depends on alotypic polymorphism of the Fcγ receptors, the isotype of PR3-ANCA or MPO-ANCA, and avidity as well as functionality of α1 AT [2]. Bearing in mind all these factors, it is clear that the ANCA titer in our patient with ISV did not correlate with BVAS (*p *> 0.05). Higher CR-P values in patients with ISV (Table 3) point to more intensive necrosis and inflammation than in indviduals with ATD-induced diseases.

Generally, ISV patients (Table 5) have a more severe course, demand more intensive immunosuppressive therapy, and have frequent relapses which we have not observed in ANCA-positive ATD-treated patients.

Therefore, patients with GD and HT should be carefully followed and monitored, not only for their thyroid status but also for the serious complications of ATD therapy. Patients with positive ANCA without evidence of vasculitis should also be carefully followed. The onset of urticaria-like vasculitis, erythrocyturia or pulmonary complaints demand immediate discontinuation of ATD.

## Conclusion

Our results show that ATDs are frequent exogenous trigger factors of ANCA-associated autoimmune diseases. In comparison with drug-induced disease, ISV patients had a severe course with more frequent renal, pulmonal and neurologic manifestations. In contrast to patients with ISV, ATD-induced ANCA-positive patients had skin manifestations more frequently, especially urticaria-like vasculitis. ATD can induce two clinically and serologically different types of diseases. Simultaneous presence of various autoantibodies characterizes ATD-induced MPO-ANCA-positive LLD with good prognosis. On the other hand, ATD-induced MPO or PR3-ANCA-positive life-threatening MPA or WG resemble ISV and were not associated with organ-nonspecific autoantibodies. Different serological profiles could help in the differential diagnosis and adequate therapeutic approach to ANCA-positive ATD-treated patients with symptoms of systemic disease.

## Abbreviations

α1 AT = alpha-1 antitrypsin; aCL = anticardiolipin antibodies; AHA = antihistone antibodies; ANA = antinuclear antibodies; ANCA = antineutrophil cytoplasmic antibodies; Anti-β2 GP I = anti-beta 2 glycoprotein I; APS = antiphospholipid syndrome; ARF = acute renal failure; ATD = antithyroid drug; BVAS = Birmingham Vasculitis Activity Score; CR-P = C reactive protein; DID = drug-induced diseases; DIV = drug-induced vasculitis; ELISA = enzyme-linked immunosorbent assay; GD = Graves' disease; HT = Hashimoto thyroiditis; IIF = indirect immunofluorescence; ISV = idiopathic systemic vasculitis; LLD = lupus-like disease; MM = methimazole; MPA = microscopic polyangiitis; MPO = myeloperoxidase; PR3 = proteinase 3; PTU = propylthiouracil; SLE = systemic lupus erythematosus; SNGN = segmental necrotizing glomerulonephritis; WG = Wegener's granulomatosis.

## Competing interests

The authors declare that they have no competing interests.

## Authors' contributions

BB-N followed and treated the patients, performed all the immunological analyses and wrote the article. MN examined the patients with skin lesions and performed and interpreted skin biopsies, and also revised the manuscript. SA followed and treated some of the patients, performed and interpreted statistical analyses and reviewed the manuscript. SZ followed the endocrinological aspects of the patients' diseases. MB performed some of the immunological analyses, followed some of the patients with idiopathic vasculitis and reviewed the manuscript.

## Figures and Tables

**Table 1:** Characteristics of the PR3/MPO-ANCA-positive patients with idiopathic and antithyroid DIDs

	Patients with idiopathic vasculitides (*n *= 56)	Patients with antithyroid DIDs (*n *= 16)
Female sex, n (%)	29 (52)	15 (94)*
Age, years	52.8 ± 10.9	31 ± 9.8**
Range	18–72	20–52
Months between first complaints and ANCA detection	3.8 ± 1.2	2.3 ± 1.1**
Graves' disease/Hashimoto thyroiditis, *n*	0	13/3
Propylthiouracil/methimazole, *n*	0	12/4

Values represent either numbers of patients (%) or mean ± standard deviation. *p < 0.01, **p < 0.001. ANCA, antineutrophil cytoplasmic antibodies; DID, drug-induced autoimmune disease; MPO, myeloperoxidase; PR3, proteinase 3.

**Table 2:** Initial clinical manifestations in PR3/MPO-ANCA-positive patients with idiopathic vasculitides and antithyroid DIDs

	Patients with idiopathic vasculitides (*n *= 56)	Patients with antithyroid DIDs (*n *= 16)
Systemic	56 (100)	16 (100)
Arthralgia/myalgia	56(100)	16 (100)
Fever >38.5°C	48 (86)	4 (25)***
Weight loss >2 kg a month	47 (84)	5 (31)***
Renal	42 (75)	3 (19)***
Acute renal failure	25 (45)	1(6)**
Proteinuria, hematuria	17 (30)	2 (12)
Lung	28 (50)	3 (19)*
Infiltrate	15 (27)	2 (12)
Nodules	10 (18)	1 (6)
hemoptysis	14 (25)	1 (6)
Pulmonary-renal syndrome	22 (39)	1 (6)*
Skin	14 (25)	10 (62.5)**
Necrosis	11(20)	2 (12)
Gangrene	3 (5)	0
Purpura	5 (9)	1 (6)
Urticaria-like vasculitis	0	5 (31) ***
Urticaria	0	2 (12.5)*
Gastrointestinal tract	7 (12)	0
Ear, nose	15 (27)	0*
Eyes	7 (12)	0
Nervous system	26 (46)	1 (6)**
Central	13 (20)	0
Peripheral	15 (27)	1 (6)

All values represent numbers of patients (%). *p < 0.05 **p < 0.01 ***p < 0.001. ANCA, antineutrophil cytoplasmic antibodies; DID, drug-induced autoimmune disease; MPO, myeloperoxidase; PR3, proteinase 3.

**Table 3:** Serological parameters in PR3/MPO-ANCA-positive idiopathic vasculitides and antithyroid DIDs

	Patients with idiopathic vasculitides (*n *= 56)	Patients with antithyroid DIDs (*n *= 16)
pANCA	28 (50)	15 (94)**
Median (1/titer)	64	256*
Range (1/titer)	32–256	64–512
MPO-ANCA	29 (52)	15 (94)**
MPO-ANCA (U/ml)	64.3 ± 22	55 ± 19
CANCA	28 (50)	1(6)**
Median (1/titer)	64	64
Range (titer)	1:32–1:256	-
PR3-ANCA	27 (48)	1 (6)**
PR3-ANCA (U/ml)	58.8 ± 17	44
ANA	3 (5)	8 (50)***
Median (1/titer)	40	80
Anti-DNA	0	1 (6)
Anti-histone	0	3(19)**
Anti-SSA	0	1(6)
aCL	6 (11)	9 (56)***
IgG	5 (9)	1 (6)
IgM	0	2 (12.5)*
IgG/IgM	1 (2)	6 (37.5)***
Anti-β2 GP I	2 (3.5)	4 (25)*
IgG	1 (2)	1 (6)
IgM	1 (2)	3 (19)*
Cryoglobulinemia	0	3 (19)**
Antimicrosomal Ab	1 (2)	13 (81)***
Antithyroglobulin Ab	1 (2)	3 (19)*
Low C4 (<0.1 g/l)	0	3 (19)**
Low α1 AT (<1.1 g/l)	13 (23)	0
High CR-P (>10 mg/l)	56 (100)	8 (50)***
CR-P mg/l	90 ± 35	46 ± 15**
Leucopenia (<3 × 10^9^/l)	1 (2)	2 (12.5)

All values represent either numbers of patients (%), or mean ± standard deviation, except median and range for *p *and cANCA titer. *p <0.05, **p < 0.01, ***p < 0.001. α1 AT, alpha-1 antitrypsin; Ab, antibodies; aCL, anticardiolipin antibodies; ANA, antinuclear antibodies; anti-β2 GP I, anti-beta 2 glycoprotein I; cANCA, cytoplasmic antineutrophil cytoplasmic antibodies; CR-P, C reactive protein; DID, drug-induced autoimmune disease; MPO, myeloperoxidase; pANCA, perinuclear antineutrophil cytoplasmic antibodies; PR3, proteinase 3.

**Table 4:** Differences in clinical and serological characteristics of PR3/MPO-ANCA positive antithyroid DIDs

	Antithyroid drug-induced vasculitides (*n *= 4)	Antithyroid drug-induced LLD (*n *= 12)
Age, years (range)	35 ± 6 (20–40)	30 ± 8 (25–52)
Female	3 (75)	12 (100)
Graves' disease	4 (100)	9 (75)
Hashimoto thyroiditis	0	3 (25)
Florid hyperthyroidism	4 (100)	3 (25)
Relapse of hyperthyroidism	2 (50)	4 (33.3)
Antimicrosomal Ab	3 (75)	10 (83.3)
Antithyroid drug therapy, months	21 ± 6	18 ± 7
Months between first complaints and ANCA detection	3 ± 1.5	2 ± 1
Propylthiouracil	2 (50)	10 (83.3)
Methimazole	2 (50)	2 (16.7)
Renal manifestations	2 (50)	1 (8.3)
Lung manifestations	3 (75)	0
Skin manifestations	0	10 (83.3)
PANCA	3 (75)	12 (100)
CANCA	1 (25)	0
MPO-ANCA (U/ml)	65 ± 20	53 ± 15
PR3-ANCA (U/ml)	44	-
ANA	0	8 (66.6)
aCL IgG, IgM	0	9 (75)
Anti-β2 GP I IgG, IgM	0	4 (33.3)
Cryoglobulinemia	0	3 (25)
Low C4 (<0.1 g/l)	0	3 (25)
High CR-P (>10 mg/l)	4 (100)	4 (33.3)
Leucopenia (<3 × l0^9^/l)	0	2 (16.7)

All values represent either numbers of patients (%), or mean ± standard deviation. Ab, antibodies; aCL, anticardiolipin antibodies; ANA, antinuclear antibodies; anti-β2 GP I, anti-beta 2 glycoprotein I; cANCA, cytoplasmic antineutrophil cytoplasmic antibodies; CR-P, C reactive protein; LLD, lupus-like disease; MPO, myeloperoxidase; PR3, proteinase 3; pANCA, perinuclear antineutrophil cytoplasmic antibodies.

**Table 5:** Disease activity and ANCA titers at presentation, treatment and outcome in study groups.

	Patients with idiopathic vasculitides (*n *= 56)			Patients with antithyroid DIDs (*n *= 16)		
	
	WG *n *= 29	MPA *n *= 23	CSSy *n *= 4	WG *n *= 1	MPA *n *= 3	LLD *n *= 12
BVAS	21.37	19.78	20.0	15	13. 0	-
cANCA, *n*	28	0	0	1	0	0
cANCA, l/titer, median	128	-	-	64	-	-
pANCA, *n*	1	23	4	-	3	12
pANCA,1/titer, median	64	256	128	-	128	256
Cyclophosphamide, *n*	29	23	4	1	2	0
Only corticosteroids *n*	0	0	0	0	1	2
cANCA after 6 months, 1/ titer, median	16*	-	-	8	-	-
pANCA after 6 months, 1/titer, median	8	16	-	-	32	128
CRF, *n*	14	10	1	0	1	0
TRF, *n*	2	6	0	0	1	0
Lethal outcome, *n*	11	2	0	0	0	0
Patients with relapses, *n*	23	22	2	0	0	0

*Median titer of 23 WG patients, six died at first presentation. BVAS, Birmingham Vasculitis Activity Score; cANCA, cytoplasmic antineutrophil cytoplasmic antibodies; CRF, chronic renal failure; CSS, Churg Strauss syndrome; DID, drug-induced autoimmune disease; LLD, lupus-like disease; MPA, microscopic polyangiitis; pANCA, perinuclear antineutrophil cytoplasmic antibodies; TRF, terminal renal failure; WG, Wegener's granulomatosis.
